# UPLC-Q-TOF-MS Study of the Mechanism of THSWD for Breast Cancer Treatment

**DOI:** 10.3389/fphar.2019.01625

**Published:** 2020-01-24

**Authors:** Xianchun Duan, Lingyu Pan, Qiuyu Bao, Daiyin Peng

**Affiliations:** ^1^Department of Pharmacy, The First Affiliated Hospital of Anhui University of Traditional Chinese Medicine, Hefei, China; ^2^Anhui Province Key Laboratory of Chinese Medicinal Formula, Synergetic Innovation Center of Anhui Authentic Chinese Medicine Quality Improvement, Anhui University of Chinese Medicine, Hefei, China

**Keywords:** Taohong Siwu decoction, breast cancer, UPLC-Q-TOF-MS, components, rats

## Abstract

Taohong Siwu decoction (THSWD) is a classic traditional Chinese medicine (TCM) prescription that is widely used in the clinical treatment of gynecological and cerebrovascular diseases. Here we used a method that coupled ultra-performance liquid chromatography to quadrupole time-of-flight mass spectrometry (UPLC-Q-TOF-MS) in which both positive and negative ion modes were established to investigate the major constituents in THSWD. A Waters ACQUITY UPLC BEH C18 column (2.1 mm×100 mm, 1.7 μm) was used to separate the aqueous extract of THSWD. The mobile phase consisted of 0.1% aqueous formic acid (A) and acetonitrile (B). Ninety-five components were identified in two different ion modes, including aromatic acids, flavones, polysaccharides, volatile oils monoterpene glycosides, aromatic cyanogenic glycosides, and others. Pathological changes in tumors and serum expression of interleukin-4 in a mouse model of breast cancer were detected after THSWD treatment. The results showed that THSWD had obvious therapeutic effects. This study establishes a material basis for the use of THSWD in the treatment of breast cancer.

## Introduction

Traditional Chinese medicine (TCM) is an important part of Chinese culture (Zhen, 2013). As the most representative and integral part of traditional Chinese culture, TCM has a long, profound history. At present, the Party and State attach great importance to TCM, which also has a far-reaching influence all over the world (Weiwei, 2017). Formulae are the main form of TCM clinical medicine (Chen and Luo, 2018). They are characterized by multiple components, which affect multiple pathways and targets, so their mechanisms of action are largely unknown (Li et al., 2012). The “Traditional Chinese Medicine Law of the People’s Republic of China” is its official implementation, marking the first time that China identified the important status, development guidelines, and supporting measures of TCM from a legal perspective and provided a legal guarantee for TCM development.

Taohong Siwu decoction (THSWD) consists of six species of medicinal herbs: Prunus persica (L.) Batsch (Taoren, TR), Carthamustinctorius L. (Honghua, HH), Angelica sinensis (Oliv.) Diels (Danggui, DG), Ligusticum chuanxiong hort (Chuanxiong, CX), Paeoniae Radix Alba (Baishao, BS), and Rehmanniaglutinosa (Gaertn.) DC. (Shudi, SD) (Ding and Zhang, 2010). THSWD was documented in “Yi Zong Jin Jian” compiled by Wu Qian in the Qing Dynasty, on the basis that the Siwu decoction enriches the blood, adds TR and HH into the blood, eliminates blood stasis, and promotes blood circulation and nourishment (Liu et al., 2015). It has been widely used in the treatment of gynecological and cerebrovascular diseases (Kim et al., 2016). Ischemic stroke, also known as cerebral infarction due to thrombosis, belongs to the category of “stroke” in TCM (Yu et al., 2007). It is an acute cerebrovascular disease characterized by sudden stupor, unconsciousness, hemiplegia, slurred speech, and speech disorder (Cheng et al., 2018).

Considering the characteristics of a TCM compound, the composition is complex. Moreover, factors like origin, cultivation, harvesting, processing, preparation, and more all influence the clinical treatment effect (Zhang, 2010). Therefore, verifying the chemical composition of TCM compounds through analysis and quality testing is particularly important. It is quite time-consuming to chemically profile TCM preparations with conventional high-performance liquid chromatography coupled with ultraviolet detection (HPLC-UV) or HPLC coupled with mass spectrometry (HPLC-MS) (Han et al., 2007; Li et al., 2014). Compared with traditional HPLC technology, which remains constant over a wider linear range, ultra-performance liquid chromatography (UPLC) has higher efficiency. Its advantages include increasing the mobile phase velocity, shortening the analysis time, and increasing throughput. The columns show superior performance, and the ultra-high pressure liquid chromatography pump offers precise gradient control, low diffusion, a low cross-contamination auto-sampler system, a high-speed detector, and comprehensive hardware and software. The peak capacity, analysis efficiency, and sensitivity are greatly improved compared with conventional HPLC, providing a good platform for the separation analysis of complex systems (Gumustas et al., 2014; Hedaya et al., 2017; Wu et al., 2017). Time-of-flight MS (TOF-MS) can provide accurate molecular mass (Zhang et al., 2018). MassHunter software can calculate the molecular formula of the test substance, so the chemical composition represented by each chromatographic peak can be directly identified with very small errors, generally at 5 ppm. This approach is widely used in the identification of compounds. In this study, UPLC-Q-TOF-MS technology was used to separate and identify the main components of THSWD.

THSWD is used to treat cerebrovascular and gynecological diseases. DG and CX have good therapeutic effects on gynecological conditions, especially breast diseases. However, there have been no reports of the study of THSWD in the treatment of breast cancer. We analyzed the components of THSWD and assessed its therapeutic effect in a mouse model of breast cancer to provide a basis for its clinical use.

## Materials and Methods

### Materials

*Prunus persica* (L.) Batsch (Taoren, TR, batch number: 17033101), *Carthamus tinctorius* L. (Honghua, HH, batch number: 17041401), *Angelica sinensis* (Oliv.) Diels (Danggui, DG, batch number: 16070501), *Conioselinum anthriscoides ‘Chuanxiong’* (syn. *Ligusticum chuanxiong* Hort) (Chuanxiong, CX, batch number: 17061601), *Paeoniae lactiflora Pall*. (Baishao, BS, batch number: 17050301), and *Rehmannia glutinosa* (Gaertn.) DC (Shudi, SD, batch number: 17042501) were purchased from Anqing Huashi Chinese Herbal Medicine Co. Ltd. (Anqing, China). All TCM materials were qualified by Professor Huasheng Peng (hspeng@126.com). All voucher specimens were deposited at the Herbarium of Anhui University of Chinese Medicine, Hefei, China (Herbarium code: ACM, voucher numbers: 17021, 17025, 17034, 17051, 17068, 17080). A Waters ACQUITY™ UPLC I-Class and a SYNAPTG2-Si MS were obtained from Waters Corporation (Milford, MA, USA). The 4T1 breast cancer cell line and fetal bovine serum (batch number: 20160720) were from Zhejiang Tianhang Biotechnology Co., Ltd. RPMI 1640 medium (batch number: ZI110516) was purchased from Hangzhou Northrend Biotechnology Co., Ltd.

### Preparation and Extraction

In this system, the herbs TR, HH, DG, SD, CX, and BS (3:2:3:4:2:3) were soaked then decocted twice with 10 vol. of boiling water for 2 h and 8 vol. of boiling water for 1.5 h. The extraction solutions were then filtered and combined. Next, 95% ethanol was added for alcohol precipitation to obtain Taohong-Siwu decoction alcohol precipitation. A small amount of alcohol solution was filtered through a 0.22-μm microporous membrane filter, and 2 μL of filtrate was used for injection analysis.

### UPLC–MS and UPLC–MS^2^ Analysis

A Waters ACQUITY UPLC BEH C18 column (2.1 mm×100 mm, 1.7 μm) was used to separate the aqueous extract of THSWD. The mobile phase was 0.1% aqueous formic acid (A) and acetonitrile (B). Chromatographic separation was performed at 35°C in this system. Gradient elution with a flow rate of 0.3 mL/min was performed as follows: 3% B at 0-2 min; 3%-8% B at 2-8 min; 8%-25%B at 8-12 min; 25%-25% B at 12-15 min; 25%-45% B at 15-16 min; 45%-90% B at 16-22 min; 90%-100% B at 22-26 min; 100% B at 26-28 min. The MS analysis was carried out with the electrospray ionization (ESI) source in both positive and negative ion modes, and leucine enkephalin was used as the accurate mass calibration solution. The desolvation gas temperature was 350°C. The flow rates of cone and desolvation gases were set at 50 L/h and 600 L/h, respectively. The capillary, cone, and extraction cone voltages were set to 3.0 and 2.5 V in positive and negative ion modes, respectively. MS^E^ was applied for MS/MS analysis with a low collision energy of 6 V and a high collision energy of 20-80V. The scan area was set at *m/z* 50-1200.

### Animal Treatment and Sample Collection

BALB/c mice (specific pathogen-free grade, 22 ± 2g), were purchased from the Laboratory Animal Center, Medical University of Anhui Province (permit number: 2016AH-031-11). They were randomly divided into six groups (10 mice each): normal; model; THSWD low-dose, middle-dose, and high-dose (L, M, and H); and cisplatin. After 4T1 cells were resuscitated, they were routinely cultured in RPMI 1640 medium containing 10% fetal bovine serum at 5% CO_2_ and 37°C. The culture medium was changed every 2 to 3 days and passaged. Cells cultured to logarithmic growth phase were collected and diluted 10 times with sterile saline, and 0.2 mL was injected into each mouse. After 48 hours, there was a small pink protrusion of about 2 mm × 2 mm, indicating successful modeling. Mice in the THSWD H, THSWD M, and THSWD L groups were treated with THSWD (25.2, 12.6, and 6.3 g/kg, respectively, three times a day) by gavage. The cisplatin group received cisplatin (0.1 mg/mL) by gavage, the normal and model groups were administered with an equivalent amount of regular saline. The dosing period was 18 days. After the last administration, blood and tumor samples were collected and animals were sacrificed by cervical vertebrae dislocation. All experiments were subject to approval by the Committee on the Ethics of Animal Experiments of Anhui University of Chinese medicine (Permit Number: LLSC20160336).

### Hematoxylin and Eosin (H&E) Staining

Tumor tissues were fixed, paraffin-embedded, and sectioned. Changes in tumor cell arrangement and pathological alterations of the tumor were observed with conventional H&E staining and analyzed using ImageJ software (National Institutes of Health, Bethesda, MD, USA).

### Interleukin (IL)-4, IL-10, IL-13, and Transforming Growth Factor (TGF)-β1 Expression

Serum was collected from mice and centrifuged at 350 g for 5 min at 4°C. Supernatants were stored at -80°C for cytokine analysis. Enzyme-linked immunosorbent assays (ELISAs) were performed to quantify the concentrations of IL-4, IL-10, IL-13, and TGF-β1, according to the manufacturer’s instructions. Optical density (OD) values were read at 450 nm, and the levels of IL-4, IL-10, IL-13, and TGF-β1 were determined based on standard curves.

### CD44, CD117, and CD133 Expression

At 24 hours after transfection, the cells were removed and washed with phosphate-buffered saline (PBS; 5 min, 3 times), fixed in methanol (-20°C, 5 min), fixed in 70% ethanol for 5 min, and washed with PBS (5 min, 3 times). They were then blocked in 1% skimmed milk powder for 30 min, and the mixture was incubated for 2 h at room temperature with primary antibody. The cells were washed with PBS, incubated with secondary antibody for 1 h, stained with 4′,6-diamidino-2-phenylindole for 2 min, washed with PBS (5 min, 3 times), mounted in DAKO medium (DAKO, Glostrup, Denmark), observed under a fluorescent microscope, and photographed.

### Data Processing

#### Establishment of a Component Analysis Database

We used the UNIFI software library management system to collect data from offline and online MS databases such as PubMed (http://www.ncbi.nlm.nih.gov/pubmed), MassBank (http://www.massbank.jp/), Chemspider (http://www.chemspider.com/), and METLIN (https://en.wikipedia.org/) and identify relevant literature on the chemical composition of THSWD. We collected the names and molecular and structural formulas to build a database of the chemical constituents of THSWD.

#### Data Acquisition and Analysis

The MS^E^ Continuum model was used to collect data and determine the chemical composition of THSWD, which was automatically identified by the UNIFI data processing system. MassLynx 4.1 software (Waters Corporation) was used to deal with the base-peak ion current patterns of positive and negative ion modes. The main chemical components were identified and confirmed by the accurate mass of fragment ions theory, relative retention time, offline and online mass spectral database, and related literature.

## Results and Discussion

### Optimization of Chromatographic Conditions and Q-TOF-MS/MS Method Development

**Figure 1:**
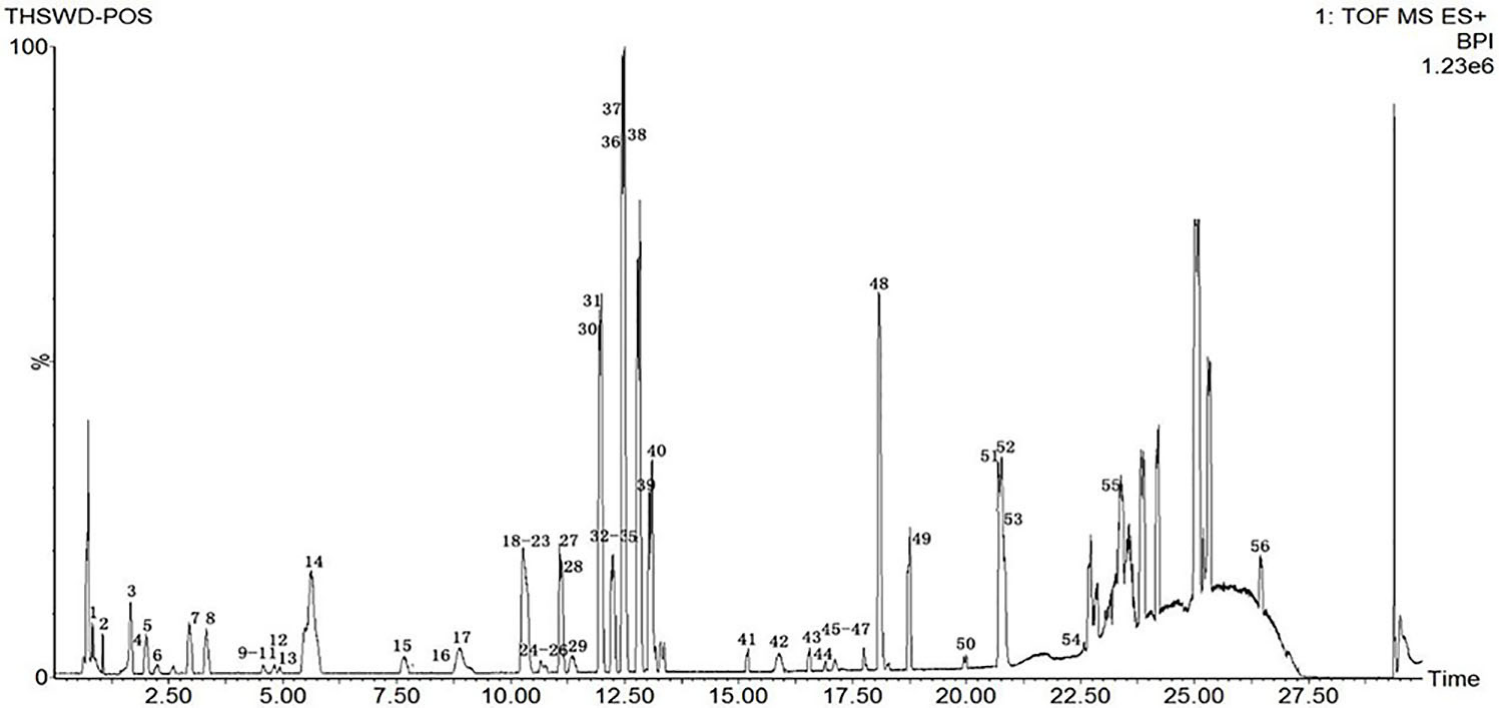
Base peak chromatograms of THSWD in positive ion mode.

**Figure 2:**
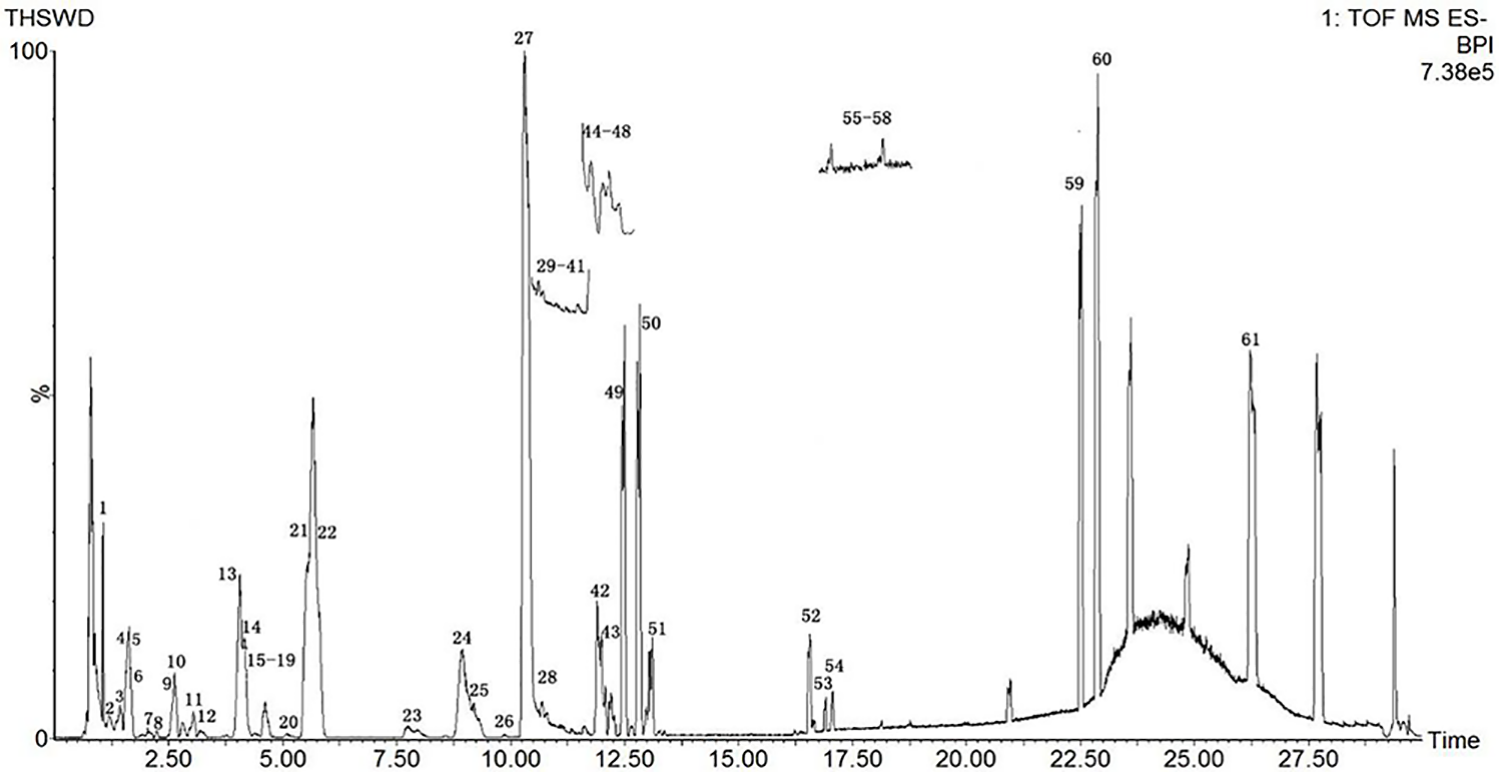
Base peak chromatograms of THSWD in negative ion mode.

Due to the diversity of TCM prescriptions, the complexity of each single herb, and the large variety of structures, it is necessary to optimize the chromatographic conditions to obtain and separate the compounds quickly and improve the resolution. The items to be improved are the mobile phase, flow rate, column temperature, and MS parameters. For the mobile phase, we chose aqueous formic acid (A) and acetonitrile (B). We set the column temperature at 35°C. The detection wavelength was based on the literature, and full-band scans were performed at 320 nm with a flow rate of 0.3 mL/min to reduce the column pressure and shorten the analytical time. The elution procedures and MS conditions were set according to section 2.3. The standard sample solution and medicinal sample solution of each ingredient in THSWD were precisely absorbed. The total ion chromatogram of the THSWD solution was obtained according to the liquid-phase and MS conditions and the injection analysis. The base peak chromatograms of THSWD in positive and negative ion modes are shown in **Figures 1** and **2**, respectively.

### Analysis of THSWD by UPLC-Q-TOF-MS and Identification of Its Main Constituents

**Figure 3:**
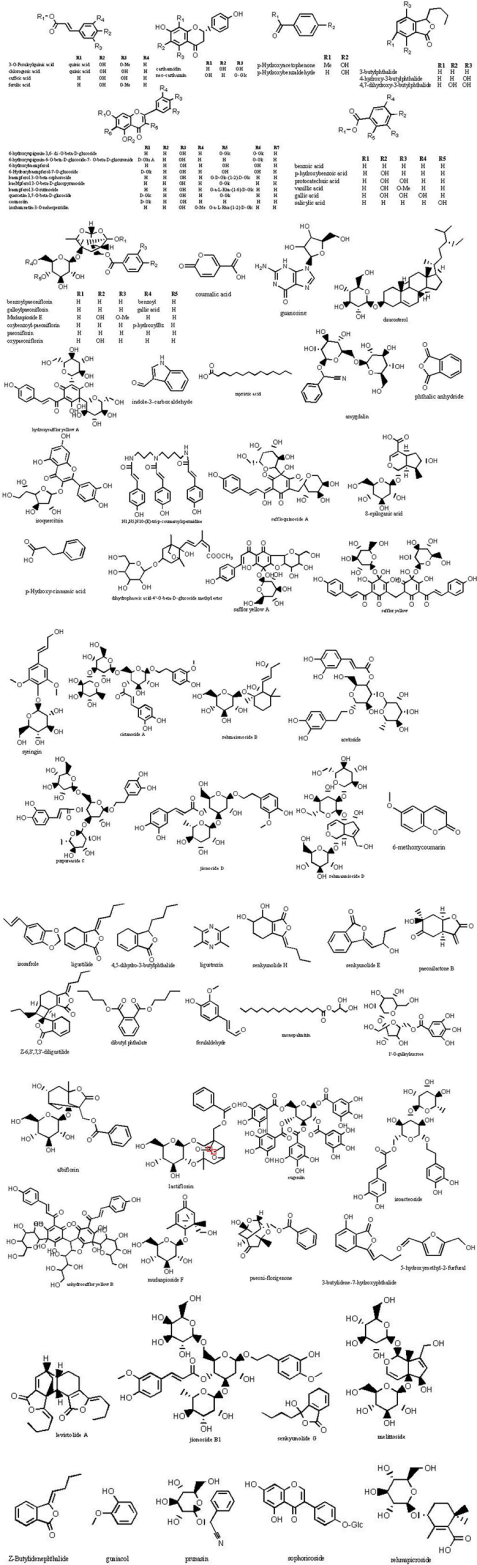
Chemical structures of the main constitutions found in THSWD.

The molecular weights of the 87 peaks extracted from the total ion chromatogram of the extract of THSWD were consistent with the data of the previously built pool, including aromatic acids, flavones, polysaccharides, volatile oils, monoterpene glycosides, aromatic cyanogenic glycosides, and others (**Table 1** and **Table 2**). Their sources were confirmed by comparing the base peak chromatograms of THSWD with its six separate herbal extracts. Standard compounds available were identified by comparing retention time with the exact mass. For compounds that were not standard, the structure was primarily based on accurate mass and tandem MS. In this study, the formulas were based on the high-precision excimer [M+H]^+^, [M+Na]^+^, [M-H]^-^, and [M+HCOO]^-^ within a mass error of 10 ppm and a partial isotopic abundance. We then searched chemical databases such as Chemspider (www.chemspider.com) and Massbank (http://www.massbank.jp) for the most rational formulas. When all isomers were matched, the structures previously reported from the six herbs of THSWD had a higher probability than the other isomers. Finally, fragment ions were used to confirm the chemical structure further. The structures underlying the main activity in THSWD are shown in **Figure 3**.

**Table 1:** Compounds identified in THSWD by UPLC-Q-TOF-MS in positive ion mode.

Peak No.	Indentify	Molecular formula	Mass error (ppm)	RT (min)	Measured mass [M+H]+/[M+Na]+	Source^a^	MS2
1	8-Epiloganic acid	C_16_H_24_O_10_	0.7	1.08	-/399.1264	S	541.1764,201.0082,167.9970
2	Guanosine	C_10_H_13_N_5_O_5_	1.8	1.52	284.0994/-	H	152.0565,135.0300
3	Gallic acid	C_7_H_6_O_5_	0.2	1.6	171.0288/-	H,B	365.1068,153.0187,98.9755
4	1’-O-galloylsucrose	C_19_H_26_O_15_	-6.2	1.98	-/517.1132	B	365.1098,261.0313,153.0174,120.0813
5	Rehmannioside D	C_27_H_42_O_20_	3.5	2.01	-/709.2186	S	365.1099,153.0174,120.0813
6	5-Hydroxymethyl-2-furfural	C_6_H_6_O_3_	-1.7	2.24	127.0388/-	S,D	181.9670,164.9865,109.0290
7	indole-3-carboxaldehyde	C_9_H_7_NO	-3.3	3.38	146.0596/-	H	188.0703,146.0596,143.0722,118.0646
8	P-Hydroxybenzoic acid	C_7_H_6_O_3_	0.1	3.76	139.039/-	H	201.0096,121.0296
9	Chlorogenic acid	C_16_H_18_O_9_	5	4	355.1041/377.0968	D	211.0240,181.0132,147.0446
10	Neo-carthamin	C_43_H_44_O_24_	1.1	4.02	451.124/-	H	313.0686,247.0266,211.0240,181.0132,147.0446
11	Safflor yellow A	C_27_H_30_O_16_	5.8	4.03	611.1642/-	H	353.1192,201.0065,105.0334
12	Syringin	C_17_H_24_O_9_	0.1	4.73	-/395.1313	H	411.1068,395.1348,161.0590
13	RehMapicroside	C_16_H_26_O_8_	-8.1	4.77	-/369.149	H	411.1068,395.1348,161.0590
14	Amygdalin	C_20_H_27_NO_11_	-0.6	5.63	458.1581/480.1474	T	496.1171,480.1473
15	Prunasin	C_14_H_17_NO_6_	0.9	7.68	-/318.0951	T	334.0687,318.0956,201.0082
16	3-O-Feruloylquinic acid	C_17_H_20_O_9_	4	8.62	369.1195/-	T	
17	paeoni-florigenone	C_17_H_18_O_6_	1.1	8.88	319.118/-	B	503.1525,105.0335
18	6-Hydroxyapigenin-6-O-β-D-glucoside-7-O-β-D-glucuronide	C_27_H_28_O_17_	2	10.28	625.1412/-	H	519.1216,323.0439,133.0644
19	Ferulaldehyde	C_10_H_10_O_3_	-2.1	10.28	179.0699/-	C,D	519.1216,503.1522,179.0697,151.0750
20	Albiflorin	C_23_H_28_O_11_	-0.1	10.28	481.1631/503.1523	B	519.1216,503.1522,500.1425,179.0697,151.0750,133.0644
21	Paeonilactone B	C_10_H_12_O_4_	-1.7	10.28	197.0805/-	B	519.1216,503.1522,500.1425,179.0697,151.0750,133.0644
22	Anhydrosafflor yellow B	C_48_H_52_O_26_	6.2	10.29	1045.2885/-	H	519.1216,503.1522,500.1425,179.0697,151.0750
23	Lactiflorin	C_23_H_26_O_10_	-0.1	10.29	463.1598/-	B	519.1216,503.1522,500.1425,179.0697,151.0750,133.0644
24	Quercetin– 3,7 – O – β – D –glucoside	C_27_H_30_O_17_	0.7	10.54	627.156/659.1488	H	303.05
25	6-Methoxycoumarin	C_10_H_8_O_3_	-2.3	10.67	177.0542/-	D	519.1263,287.0570,177.0547,171.9834,89.0389
26	paeoniflorin	C_23_H_28_O_11_	0.7	10.75	-/503.1527	B	503.1530,201.0068,163.0387
27	6-hydroxykaempferol	C_15_H_10_O_7_	2.9	11.29	303.0503/-	T,H	491.2935,476.3285,453.3431
28	Cistanoside A	C_36_H_48_O_20_	-1.5	11.34	-/823.2619	D	693.1618,635.1593,351.1442,177.0546,145.0277
29	Benzoic acid	C_7_H_6_O_2_	7.2	11.36	-/145.027	B	351.1442,289.0703,177.0546
30	Galloylpaeoniflorin	C_30_H_32_O_15_	1.6	11.91	633.1752/655.1644	B	655.1640,153.0181,147.0443
31	Saffloquinoside A	C_27_H_30_O_15_	1	12.04	595.1663/617.159	H	503.1527,287.0553,177.0544
32	Isosafrole	C_10_H_10_O_2_	-1	12.13	163.0752/-	C,D	91.0546
33	Isorhamnetin-3-O- nehesperridin	C_28_H_32_O_16_	1.2	12.18	625.177/657.1698	C	814.5436,317.0659,302.0421,301.0351
34	6-hydroxyapigenin	C8H4O3	-4.5	12.19	287.0537/-	D	673.4566,579.2918,363.1112,317.1154,301.1413,160.9908,149.0233
35	Acetoside	C_29_H_36_O_15_	4.3	12.26	-/647.1974	S	287.0555,163.0388
36	Kaempferol 3-O-rutinoside	C_27_H_30_O_15_	6.5	12.4	595.1696/-	T,H	519.1306,503.1549,317.0680,302.0432,205.0721,181.0127,140.0510
37	cosmosiin	C_21_H_20_O_10_	2.6	12.4	433.114/-	C	301.1412,149.0232
38	Carthamidin	C_15_H_12_O_6_	0.6	12.59	289.0708/-	H	153.0181,147.0443
39	Senkyunolide H	C_12_H_16_O_4_	-1.8	13.13	-/247.0936	C	1165.1875,1045.4538,926.4874,789.3951,712.7139,601.8298
40	4-hydroxy-3-butylphthalide	C_12_H_14_O_3_	-0.7	13.15	207.1014/-	C,D	247.0952,147.0446,91.0545
41	ferulic acid	C_10_H_10_O_4_	4.8	15.2	-/217.0482	C,D	279.0198,163.0395,92.0262
42	safflospermidine A	C_34_H_37_N_3_O_6_	1.8	15.81	584.2766/606.2693	H	204.1032,147.0438
43	oxybenzoyl-paeoniflorin	C_30_H_32_O_12_	1.3	16.55	-/607.1794	B	607.1794,249.0760,105.0335
44	Benzoylpaeoniflorin	C30H32O12	1.1	16.65	585.1973/-	B	133.0663
45	Jionoside B1	C_37_H_50_O_20_	-5.9	17.09	-/837.2738	S	228.2324,107.0853,105.0695
46	Z-Butylidenephthalide	C_12_H_12_O_2_	-0.8	17.15	189.0908/-	C,D	608.1815,219.0692,128.0629
47	Ligustilide	C_12_H_14_O_2_	-2.9	17.22	191.1061/-	C,D	670.3756,447.2399,355.2629,181.1199,165.0908,92.0252
48	4,5-dihydro-3-butylphthalide	C_12_H_16_O_2_	4	18.27	193.1231/-	C	242.2845
49	3-Butylphthalide	C_12_H_14_O_2_	1.1	19.12	191.1069/-	C,D	191.1081,128.0624,115.0542
50	Levistolide A	C_24_H_28_O_4_	0.6	20.62	381.2063/403.199	D	419.1643,311.2948,256.2643
51	Dibutyl phthalate	C_16_H_22_O_4_	2.7	20.75	-/301.1418	C	787.52097,743.4928,337.3110
52	3-Butylidene-7-hydroxyphthalide	C_12_H_12_O_3_	0.3	20.77	205.086/227.0787	C	301.1412,149.0232
53	Z-6,8’,7,3’- diligustilide	C_24_H_28_O_4_	-0.6	20.82	381.2058/-	C	510.3556,492.3458,473.2644,327.2904,191.1072
54	Eugeniin	C_41_H_30_O_26_	6.5	22.3	-/961.098	H	283.2633,281.2476
55	Daucosterol	C_35_H_60_O_6_	-9.4	23.27	-/599.4226	H,C,D	283.2632,243.1214,95.0858
56	Ligustrazin	C_8_H_12_N_2_	-1.9	26.77	137.1071/-	C	302.0432,205.0720,181.0127

a H, T, D, C, B, S indicate Prunus persica (L.) Batsch, Carthamus tinctorius L., Angelica sinensis (Oliv.) Diels, Conioselinum anthriscoides ‘Chuanxiong’ (syn. Ligusticum chuanxiong Hort), Paeoniae lactiflora Pall., and Rehmannia glutinosa (Gaertn.) DC, respectively.

**Table 2:** Compounds identified in THSWD by UPLC-Q-TOF-MS in negative ion mode.

Peak No.	Indentify	Molecular formula	Mass error (ppm)	Observed RT (min)	Measured mass[M-H]^-^/[M+HCOO]^-^	Source^a^	MS^2^
1	8-epiloganic acid	C_16_H_24_O_10_	-2.1	1.09	^_^/421.1343	S	499.1666,290.0882,128.0352
2	Guanosine	C_10_H_13_N_5_O_5_	0.2	1.53	282.0844/-	H	377.0849,341.1088,282.0841,161.0454,150.0421
57	Coumalic acid	C_6_H_4_O_4_	1	1.61	^_^/185.0093	H	169.0142
3	gallic acid	C_7_H_6_O_5_	-0.3	1.62	169.0142/^_^	H,B	169.0142
6	5-Hydroxymethyl-2-furfural	C_6_H_6_O_3_	-0.5	1.62	125.0244/^_^	S,D	169.0142
58	6-hydroxykaempferol-7-O-β-D- glucoside	C_21_H_20_O_12_	0.2	1.73	463.0883/^_^	T,H	329.0867,302.0675,287.0878,134.0475
5	Rehmannioside D	C_27_H_42_O_20_	3.2	2.06	685.2293/731.2275	S	493.1197,331.0622,313.0567,169.0145,164.0721
59	Melittoside	C_21_H_32_O_15_	0.4	2.17	523.1743/569.1725	H	491.1190,448.1490
7	indole-3-carboxaldehyde	C_9_H_7_NO	1.8	2.45	-/190.0513	H	589.1873,379.1206
60	protocatechuic acid	C_7_H_6_O_4_	0.9	2.65	153.0195/^_^	H,C	512.1358,510.1380,474.1612,150.0560
61	Mudanpioside F	C_16_H_24_O_8_	-1	3.37	343.1467/389.1449	B	379.1148
62	salicylic acid	C_7_H_6_O_3_	-0.5	3.78	137.0244/-	S,D	193.0515,134.0373
9	Chlorogenic acid	C_16_H_18_O_9_	-0.9	4.04	353.0875/-	H	325.0712,191.0561,119.0503
63	hydroxysafflor yellow A	C_27_H_32_O_16_	1.3	4.06	611.1626/-	H	503.1185,491.1189,403.1027,191.0561
64	Guaiacol	C_7_H_8_O_2_	-0.4	4.37	123.0451/^_^	C	495.1511,167.0360
65	Vanillic acid	C_8_H_8_O_4_	-0.5	4.38	167.0349/-	D	493.1197,331.0622,313.0567,169.0145,164.0721
66	oxypaeoniflorin	C_23_H_28_O_12_	-0.2	4.41	495.1507/541.158	H	465.1368,177.0568,167.0359,137.0244
12	syringin	C_17_H_24_O_9_	-1.9	4.76	-/417.1394	H	163.0393,119.0501
13	RehMapicroside	C_16_H_26_O_8_	-0.9	4.8	345.1624/391.1606	H	463.0886,301.0346,271.0257,151.0042,116.9285
67	caffeic acid	C_9_H_8_O_4_	-0.2	5.09	179.035/^_^	D,C	135.04438
24	Quercetin– 3,7 – O – β – D –glucoside	C_27_H_30_O_17_	-1	5.45	625.1404/-	H	463.0898,407.0280,301.0361,299.0217
68	p-Hydroxybenzaldehyde	C_7_H_6_O_2_	-1.1	5.46	121.0294/^_^	H	625.1423,605.0494,480.1163,463.0898,301.0361,299.0218,146.9627
15	prunasin	C_14_H_17_NO_6_	-1.4	7.73	294.1051/340.1033	T	357.0950,332.0729,330.0740
69	p-Hydroxy-cinnamic acid	C_9_H_10_O_2_	-1	8.56	163.0399/-	H	609.1463,435.0922,285.0407,193.0511,134.0371,133.0294
16	3-O-Feruloylquinic acid	C_17_H_20_O_9_	-0.9	8.71	367.1031/^_^	T	
20	albiflorin	C_23_H_28_O_11_	-0.7	9.2	479.1628/525.161	B	542.1508,517.1288,515.1318
70	Mudanpioside E	C_24_H_30_O_13_	0.2	10.31	525.1615/^_^	B	517.1305,515.1323,449.1447,327.1084,165.0555
18	6-hydroxyapigenin-6-O-β-D-glucoside-7-O-β-D-glucuronide	C_27_H_28_O_17_	-0.6	10.43	623.1268/^_^	H	525.1612,517.1305,515.1323,449.1447,327.1084,121.0294
71	6-hydroxyapigenin-3,6- di -O– β – D –glucoside	C_27_H_30_O_17_	1.2	10.56	625.1417/^_^	H	625.1414,609.1461,463.0871,462.0796,301.0347,271.0248,243.0307,139.0042
41	Ferulic acid	C_10_H_10_O_4_	0.1	10.69	193.0507/-	H,C,D	193.0511,134.0370,133.0294,132.0210
72	Purpureaside C	C_35_H_46_O_20_	2.9	10.78	785.2533/-	S	623.2193,495.1525,193.0507,161.0244,137.0246
73	safflor yellow	C_27_H_30_O_15_	0.9	11.05	609.1466/-	H	301.0350,300.0277
74	ISOQUERCITRIN	C_21_H_20_O_12_	-0.8	11.14	463.0878/-	T,H	271.0248,243.0299,116.9286
75	kaempferol 3-O– β-sophoroside	C_27_H_30_O_16_	0.1	11.21	609.1462/-	T,H	609.1462,284.0325,227.0352,151.0040
76	Dihydrophaseic acid-4’-O-β-D-glucoside methyl ester	C_22_H_34_O_10_	-0.8	11.25	^_^/503.213	H	610.1502,493.1855,430.0922,361.1669,283.0260,145.0286
28	Cistanoside A	C_36_H_48_O_20_	3.3	11.34	799.2692/845.2765	D	799.2694,624.2226,623.2197,193.0509,175.0399,161.0245
11	safflor yellow A	C_27_H_30_O_16_	-1.5	11.38	611.1608/657.1681	H	609.1464,301.0351,300.0277
77	Rehmaionoside B	C_19_H_34_O_8_	-1.4	11.83	^_^/435.223	S	695.5073,595.1719,425.1937,389.0877,347.0778,149.0247
39	Senkyunolide H	C_12_H_16_O_4_	0.7	11.91	223.0977/-	C,D	631.1673,169.0140,161.0242
30	galloylpaeoniflorin	C_30_H_32_O_15_	0.9	11.92	631.1674/^_^	B	631.1673,613.1566,399.0927,313.0564,271.0464,169.0140,161.0242
10	neo-carthamin	C_43_H_44_O_24_	-1.1	11.93	449.1084/^_^	H	631.1673,313.0564,271.0464,169.0140,161.0242
36	Kaempferol 3-O-rutinoside	C_27_H_30_O_15_	0.7	12.05	593.1516/^_^	T,H	593.1513,285.0399,284.0327,255.0230,227.0348,175.0399
45	Jionoside B1	C_37_H_50_O_20_	2.6	12.08	813.2844/859.2916	S	593.1513,285.0399,284.0327,255.0230,227.0348,175.0399
78	p-Hydroxyacetophenone	C_8_H_8_O_2_	-1.2	12.13	135.045/^_^	H	801.1863,637.1470,467.2142,146.9655
37	cosmosiin	C_21_H_20_O_10_	-1.8	12.14	431.0976/^_^	H	801.1863,626.1655,146.9655
33	Isorhamnetin-3-O- nehesperridin	C_28_H_32_O_16_	1.6	12.19	623.1627/-	C	623.1624,315.0504,243.0300
79	kaeMpferol 3-O--D-glucopyranoside	C_21_H_20_O_11_	-1	12.27	447.0928/-	T,H	284.0330,227.0351,161.0242,133.0295
80	Isoacteoside	C_29_H_36_O_15_	-0.2	12.27	623.1985/^_^	T	574.1599,424.1033,364.0819,311.0931,161.0238,133.0197
81	Sophoricoside	C_21_H_20_O_10_	-0.5	12.41	431.1054/477.1036	H	611.1398,403.0806,287.0566,162.8394,119.0501
82	Jionoside A	C_30_H_38_O_15_	0	13.12	637.2138/-	D	451.3276,347.0761,174.9555,161.0247
23	lactiflorin	C_23_H_26_O_10_	-0.2	13.26	461.1525/507.1507	B	539.1899,497.1225,237.0770,121.0299
44	benzoylpaeoniflorin	C_30_H_32_O_12_	1.8	16.55	583.1905/629.1887	B	619.1593,121.0294
43	oxybenzoyl-paeoniflorin	C_30_H_32_O_12_	0.7	16.82	583.19898/629.188	B	134.0371,121.0294
83	4,7-dihydroxy-3-butylphthalide	C_12_H_14_O_4_	0.6	17.17	221.0821/^_^	C	290.0673,205.0884,161.0975
84	Senkyunolide G	C_12_H_16_O_3_	-0.6	17.24	207.1025/^_^	C	265.0631,221.8433,163.1134
40	4-hydroxy-3-butylphthalide	C_12_H_14_O_3_	-0.8	17.38	205.0869/-	C	237.0929,134.0374
85	senkyunolide E	C_12_H_12_O_3_	-0.5	17.73	203.0713/^_^	C	203.07211,145.0288,132.0230
48	4,5-dihydro-3-butylphthalide	C_12_H_16_O_2_	-4.7	17.91	191.1069/-	C	925.4829,701.4286,447.1355
86	Myristic acid	C_14_H_28_O_2_	-0.2	22.43	227.2016/-	H	285.1609,227.2017
87	monopalmitin	C_19_H_38_O_4_	-1.6	22.97	-/375.2746	H,C	429.1942,99.9258
56	Daucosterol	C_35_H_60_O_6_	0.2	26.31	-/621.4373	S	255.2330,96.9601

a H, T, D, C, B, S indicate Prunus persica (L.) Batsch, Carthamus tinctorius L., Angelica sinensis (Oliv.) Diels, Conioselinum anthriscoides ‘Chuanxiong’ (syn. Ligusticum chuanxiong Hort), Paeoniae lactiflora Pall., and Rehmannia glutinosa (Gaertn.) DC, respectively.

#### Phenolic Acids

Seven phenolic acids of THSWD were identified in positive ion mode. Most from *Conioselinum anthriscoides ‘Chuanxiong’* and *Angelica sinensis* (Oliv.) Diels were ferulic acid, chlorogenic acid, caffeic acid, and vanillic acid, and gallic acid was from *Paeonia lactiflora* Pall. The retention time and MS information of each chemical component in THSWD were determined by UPLC-MS, and the chemical composition was confirmed by combining the extracted ion current map with data in the relevant literature. Compound 41 showed the t_R_ at the 15.2-min peak obtained in ESI^-^mode at the *m/z* 194.059 ion peak. The literature (Lin et al., 1998; Huang and Song, 2001; Lu et al., 2004) reports that the relative molecular mass of ferulic acid in *Conioselinum anthriscoides ‘Chuanxiong’* (syn. *Ligusticum chuanxiong* Hort) was 194. It was speculated that *m/z* 194.059 was its quasi-molecular ion peak, and MS^2^ analysis was performed on *m/z* 193, which were *m/z* 279.0198, 163.0395, 92.0262, and other fragment peaks. According to the elemental composition analysis, the molecular formula is C_10_H_10_O_4_. It showed a theoretical value of the relative molecular mass of 193.0506 and a measured value of 194.059. This peak adds Na in ESI+ mode to obtain the ion peak with *m/z* 217. MS^2^ analysis was performed on *m/z* 217 to obtain fragment peaks such as *m/z* 123 and 151. According to the elemental composition analysis, the molecular formula was C_10_H_10_O_4_, and the theoretical value of the relative molecular mass was 217.0471. The actual value was 217.0475. Based on this, the compound was assumed to be ferulic acid.

**Figure 4:**
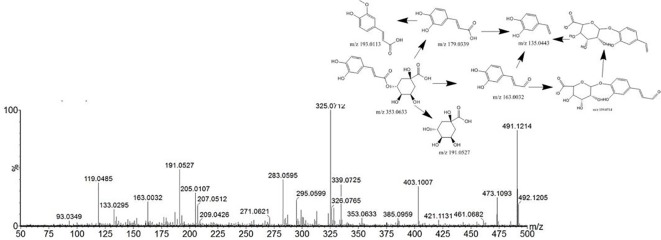
Tandem mass spectra and possible fragment pathways of chlorogenic acid in positive ion mode.

Compound 9 showed a [M+H] ^+^ion at *m/z* 355.1041, and more abundant ions in the MS^2^ spectrum were *m/z* 211.0240, 181.0132, and 147.0446. It was identified as chlorogenic acid from *Angelica sinensis* (Oliv.) Diels. The MS^2^ spectrum and possible fragmentation pathways of chlorogenic acid are depicted in **Figure 4**. Compound 67 had a retention time of 5.09 min, and the characteristic ion fragment is 179[M-H]^-^. It can be deduced that the molecular formula may be C_9_H_8_O_4_. In the further MS cleavage process, its quasi-molecular ion removes 1 molecule of CO_2_ to form a characteristic fragment ion *m/z* 135.04438 [M-H-CO2]-. By comparison with the standard spectrum, it was confirmed to be caffeic acid.

In negative ion mode, the retention time of compound 3 was 1.62 min, and the peak of its excimer ion was *m/z* 169.0142 [M -H]^-^. It can be deduced that the molecular formula may be C_7_H_6_O_5_; the error is 0.3. In positive ion mode, the excimer ion peak was *m/z* 171.0227 [M + H]^+^ with an error of 0.2. In the subsequent MS cleavage process, the quasi-molecular ion removed one molecule of CO_2_ to form the characteristic fragment ion *m/z* 125.0215 [M-H-CO_2_]^-^, indicating that the molecular structure may contain carboxylic acid groups. The related literature (Sun et al., 2009; Jiang et al., 2011; Liu et al., 2017) is consistent with the main characteristics of gallic acid, so that was inferred to be the identity of compound 3.

#### Flavones

Most of the six drugs of THSWD contain flavonoids that have a high response in positive and negative ion modes, but some compounds only respond in negative ion mode with [M-H]^-^ and [M+HCOO]-. To facilitate the discussion of MS cleavage of different flavonoids in THSWD, the nomenclature of fragment ions for flavonoids was used (Ma et al., 1997; Feng et al., 2017). The main active ingredient in *Carthamus tinctorius* L. is safflor yellow. Viewing the information on the mass spectrum of compound 63, it showed a molecular weight at 612.1698. The fragment *m/z* 611[M-H]^-^ was the excimer ion peak. By reading the literature (Ning et al., 2012; Zhang et al., 2014), it was inferred to be hydroxy safflor yellow A with a molecular formula of C_27_H_32_O_16_. At the same time, compound 11 was identified as safflor yellow A. Compound 10 was identified as neocarthamin.

Some flavonoids are mostly present in the form of flavonoid glycosides. By comparison with the reference substance, it was determined that compounds 24, 35, 19, and 12 were quercetin, apigenin, kaempferol, and isorhamnetin, respectively. The retention time and lysis of the reference substance were in accordance with these inferences.

#### Polysaccharides

Polysaccharides are one of the main active ingredients of THSWD and are mainly from *Angelica sinensis* (Oliv.) Diels and *Rehmannia glutinosa* (Gaertn.) DC. Compound 4 had a retention time of 1.98 min, the excimer ion peak was *m/z* 493 [M-H]^-^, the fragment *m/z* 331 was formed as an excimer ion peak, and a glucose residue (162 u) was formed. The characteristic fragment was *m/z* 169. The ions were derived from galloyl anions, and fragment ions of *m/z* 125 were generated after the gallic acyl anion underwent hydroxyl alpha cleavage to lose one molecule of CO_2_ (44 u). According to the literature, the three isomers 1’-O-galloyl sucrose, 6’-O-galloyl sucrose, and 6-O-galloyl sucrose exist in the white peony, but from the total ion chromatogram A *m/z* 493 was extracted, and only one peak appeared at 4.549 min. Therefore, it is presumed that compound 4 is galloyl sucrose, but the linking position of its sugar needs to be confirmed.

#### Volatile Compounds

The major volatile oils were from *Angelica sinensis* (Oliv.) Diels and *Conioselinum anthriscoides ‘Chuanxiong’* (syn. *Ligusticum chuanxiong* Hort). Compound 47 showed an [M+H]^+^ ion at *m/z* 191.1061. The literature (Wang and Han, 2011) has reported that the relative molecular mass of ligustilide in *C. anthriscoides ‘Chuanxiong’* was 190. It was speculated that *m/z* 191 was its quasi-molecular ion peak. MS^2^ analysis was performed on *m/z* 191, which yielded fragment peaks such as m/z 670, 447, and 355. It showed a retention time of 17.22 in positive ion mode. According to the elemental composition analysis, the molecular formula was C_12_H_14_O_2_, the theoretical value of the relative molecular mass was 191.1061, and the measured value was 191.1061. Based on this, it was assumed that the compound was ligustilide.

**Figure 5:**
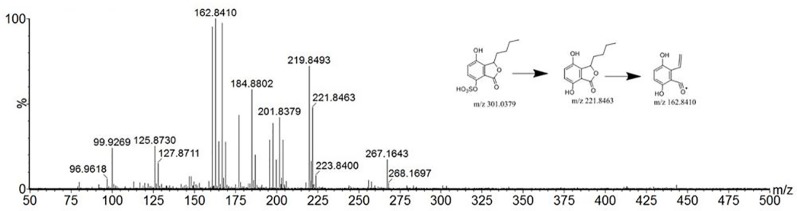
Tandem mass spectra and possible fragment pathways of 4-hydroxy-3-butyl-benzoquinone in positive ion mode.

Compound 40 showed a retention time of 13.15 min in positive ion mode, with an [M+H]+ ion at *m/z* 207.1014. The peak with t_R_ of 17.38 min yielded an *m/z* 205.0869 ion peak in the ESI^-^. The literature (Yang et al., 2007) reports that the relative molecular mass of 4-hydroxy-3-butyl- benzoquinone in *C. anthriscoides ‘Chuanxiong’* is 206. According to the MS^2^ results, it was assumed that the compound was 4-hydroxy-3-butyl-benzoquinone. The MS^2^ spectrum and possible fragmentation pathways are depicted in **Figure 5**.

#### Monoterpene Glycosides

**Figure 6:**
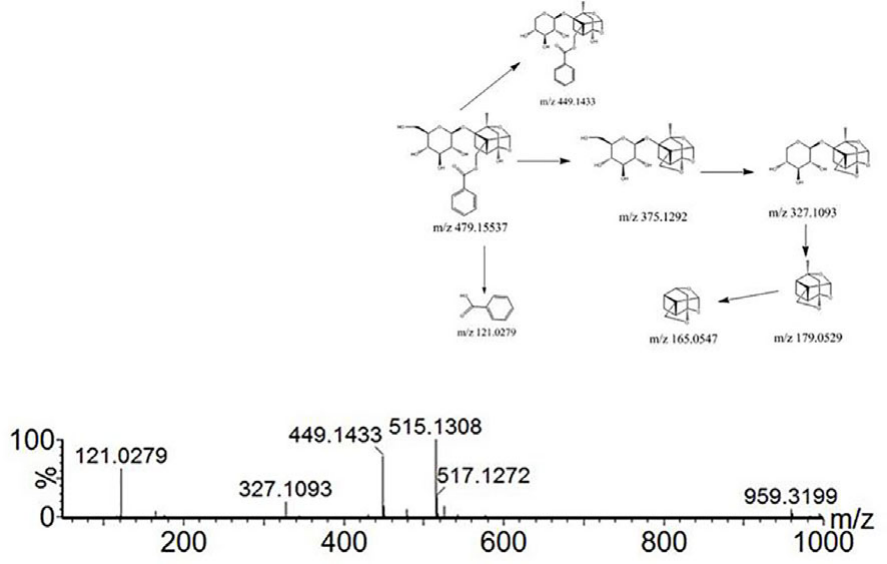
Tandem mass spectra and possible fragment pathways of paeoniflorin in positive ion mode.

Compound 21 showed a retention time of 10.28 min. The fragment ions in the mass spectrum were *m/z* 519.1216, 503.1522, 500.1425, 179.0697, 151.0750, and 133.0644, and the excimer ion peak was *m/z* 479 [M-H]^-^. The MS2 spectrum and possible fragmentation pathways are depicted in **Figure 6**. Fragment ion *m/z* 525 formed for the molecular ion peak plus one molecule of formic acid (46 u). Due to the presence of a lactone ring in the structure of paeoniflorin, its acyloxy groups can be broken on both sides, losing one molecule of CO_2_ (44 u) at m/z 435. The fragment *m/z* 357 was formed as the excimer peak and lost one molecule of benzoic acid (122 u), and *m/z* 121 was the missing one of the benzoic acid fragment ions. A combination of fragment *m/z* 481 [M+H]^+^ and fragment *m/z* 503.1522 [M+Na]^+^ appeared in positive ion mode, indicating that compound 21 is paeonolide B.

For compound 26, the retention time was 12.393 min. The fragment ions in the mass spectrum had *m/z* 525, 449, 327, and 165. The molecular ion peak was *m/z* 479 [M+H]^-^, and the fragment ion *m/z* 525 was the molecular ion peak plus a molecule of formic acid (46 u). *m/z* 449 is the molecular ion peak (*m/z* 479 [MH]-) that loses a molecule of formaldehyde (30 u) on the 6-carbon residue of the glucosyl group, while the *m/z* 449 fragment ion further loses a molecule of benzoic acid (122 u) to form the m/z 327 fragment ion. *m/z* 165 is a fragment of the decyl alkyl skeleton structure, and since the decane skeleton is connected with a benzoyl substituent, *m/z* 121 fragments were generated here. Combining the fragments *m/z* 503 [M + Na]^+^ appearing in positive ion mode, the literature suggests that compound 26 is peoniflorin.

Compounds 43 and 44 showed the same molecular weight at 584 Da and almost consistent tandem MS behavior with paeonolide. The fragment ions in the mass spectrum were *m/z* 629, 553, and 431. The fragment ion *m/z* 629 was the molecular ion peak (*m/z* 583 [M-H]^-^) plus one molecule of formic acid (46 u), and m/z 553 was the molecule. The ion peak (m/z 583 [M-H]^-^) loses the formation of a molecule of formaldehyde (30 u) on the 6-carbon residue of the glucosyl group, and the fragment ion *m/z* 553 further loses a molecule of benzoic acid (122 u), resulting in *m/z* 431 fragment ions. Positive ion mode yielded m/z 585[M + H]^+^ and *m/z* 607 [M + Na]^+^, which identified compound 44 as benzoyl glycoside according to the literature.

#### Aromatic Cyanogenic Glycosides

Most of the common cyanogenic glycosides have the same skeleton structure. According to the substituents on the cyanohydrin derivatives, they can be divided into two categories: aliphatic and aromatic. A given species of plant generally contains only one or two cyanogenic glycosides, such as Rosaceae. Compounds 14 and 15 were all derived from dry mature seeds of the Rosaceae plant *Prunus persica* (L.) Batsch or *Prunus davidiana* (Carr.) Franch.

#### Others

THSWD also contains human essential amino acids, vitamins, and other ingredients.

#### H&E

**Figure 7:**
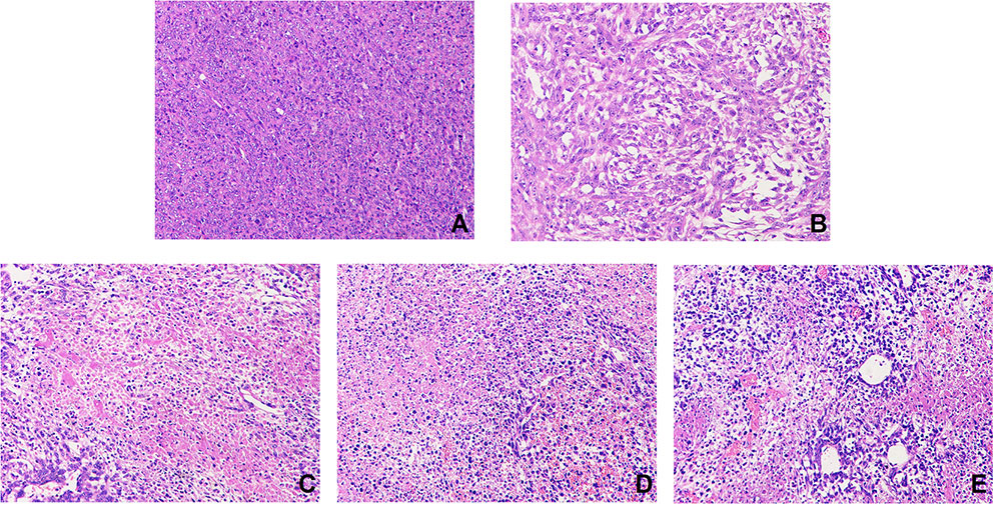
Effect of THSWD on tumor cells in mice. Sections were stained with H&E. Magnification, ×200; scale bars, 50 μm. Model group **(A)**, cisplatin group **(B)**, THSWD H group **(C)**, THSWD M group **(D)**, THSWD L group **(E)**.

H&E stained samples for each group are shown in **Figure 7**. Tumor cells in the model group were arranged closely, with less necrosis and a large nuclear-to-plasma ratio. The cell morphology was abnormal, and there was more vascular filling. Compared with the model group, tumor cells in the THSWD groups and cisplatin group showed large areas of necrosis with obvious cell debris. The necrotic cells were loosely arranged with large gaps. The results indicate that THSWD exerts a destructive effect on mouse breast cancer cells. This is the first evidence that THSWD has a significant therapeutic effect on breast cancer tumor cells.

### IL-4, IL-10, IL-13, and TGF-β1 Expression

To explore the mechanism of THSWD on breast cancer, levels of IL-6, IL-8, and IL-1β were measured by ELISA (**Table 3**). IL-5 was significantly increased in the model group. After treatment with THSWD or cisplatin, IL-5 was significantly reduced. The results indicate that THSWD may regulate immune function in breast cancer mice.

**Table 3:** IL-4, IL-10, IL-13, and TGF-β1 expression.

Group	IL-4(pg/ml)	IL-10(pg/ml)	TNF-a	TGF-β1(ng/ml)
N	366.21±38.07	1097.42±106.03	1154.64±174.62	402.80±31.10
M	336.18±51.74^##^	1028.10±43.31^#^	1657.05±268.54^##^	445.98±26.76^#^
C	506.78±64.39^**^	1121.88±105.69^*^	1338.12±56.04^*^	413.13±11.27^*^
H	354.10±44.88	1112.76±102.59^**^	1336.89+344.15^*^	436.98±34.06
M	386.41±45.83^**^	1175.52±115.75^*^	1607.49±170.73	400.86±40.15^*^
L	343.69±40.53	1048.27±143.15	1335.96±296.74^*^	424.23±13.06

Data are presented as mean ± SD. n = 8 per group. **P < 0.01 vs. control group,

^##^P < 0.01 vs. model. H, high-dose; M, middle-dose; L, low-dose; N, normal; M, model.

### CD44, CD117, and CD133 Expression

**Figure 8:**
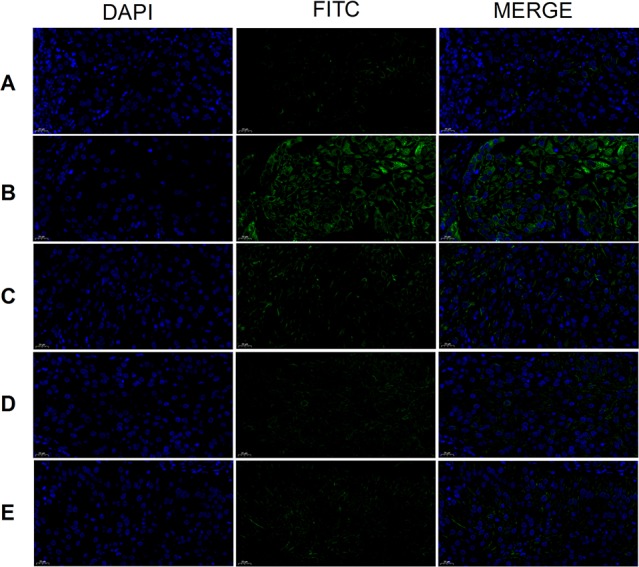
CD44 expression (×400). Model group **(A)**, cisplatin group **(B)**, THSWD H group **(C)**, THSWD M group **(D)**, THSWD L group **(E)**.

Tumor cell CD44 expression was highest in the model group and lowest in the cisplatin group (**Figure 8**). There was no obvious difference between the three THSWD dose groups. The results showed that cisplatin and THSWD significantly inhibited CD44 expression in tumor cells of breast cancer mice.

**Figure 9:**
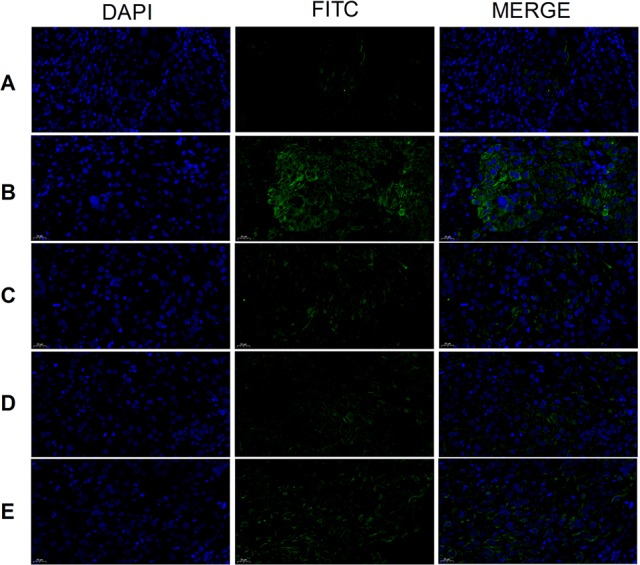
CD117 expression (×400). Model group **(A)**, cisplatin group **(B)**, THSWD H group **(C)**, THSWD M group **(D)**, THSWD L group **(E)**.

CD117 expression was highest in the tumor cells of the model group and lowest in the cisplatin and THSWD middle-dose groups (**Figure 9**). The results showed that cisplatin and THSWD significantly inhibited CD117 expression in tumor cells of breast cancer mice.

**Figure 10:**
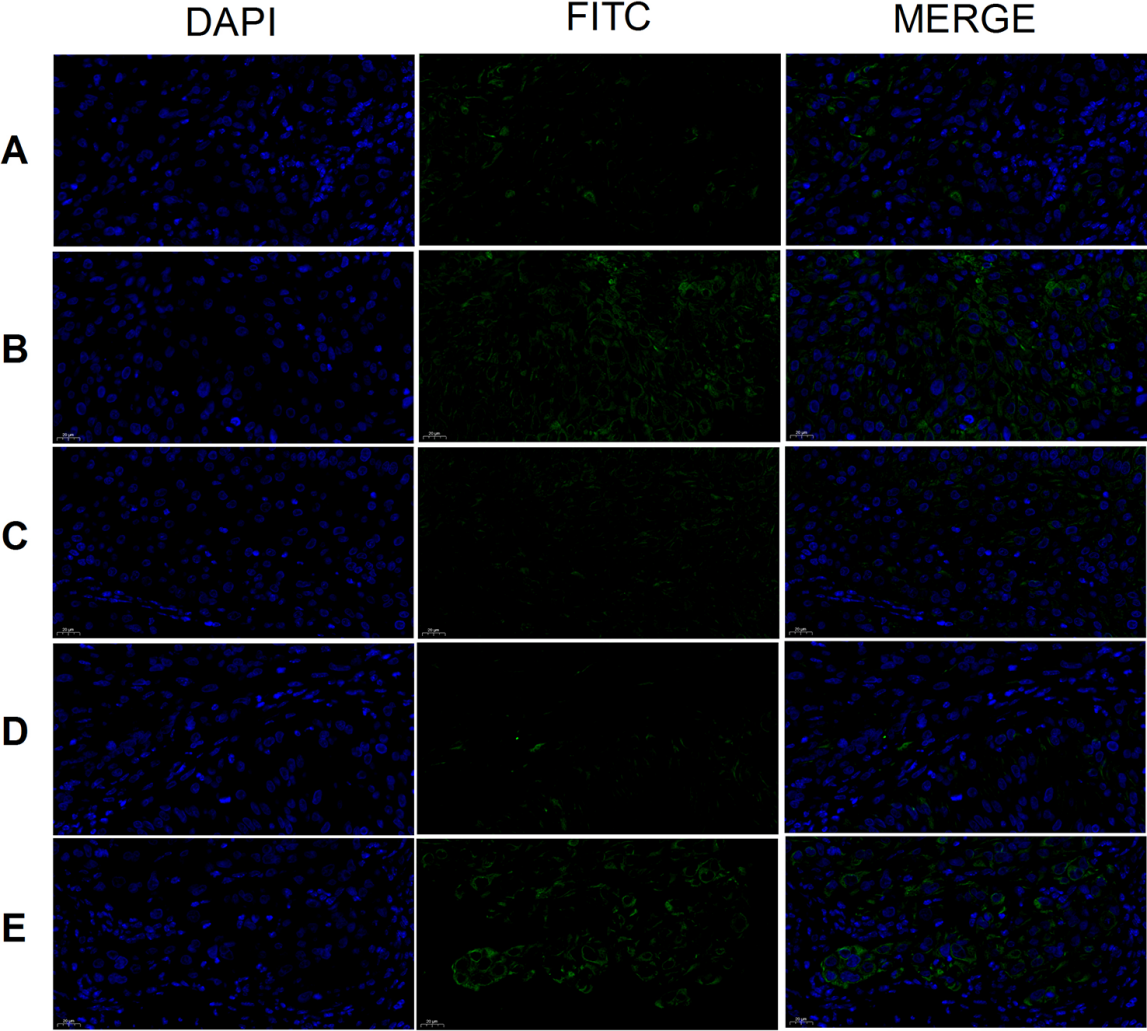
CD133 expression (×400). Model group **(A)**, cisplatin group **(B)**, THSWD H group **(C)**, THSWD M group **(D)**, THSWD L group **(E)**.

The expression of CD133 was highest in tumor cells of the model group and lowest in the cisplatin group (**Figure 10**). Results were similar for the THSWD high-, middle-, and low-dose groups. Both cisplatin and THSWD significantly inhibited CD133 expression in tumor cells of breast cancer mice.

## Conclusion

In this study, UPLC-Q-TOF-MS was used for the full chemical characterization of THSWD in both positive and negative ion modes. A total of 87 compounds were identified in 32 minutes, including aromatic acids, flavones, polysaccharides, volatile oils, monoterpene glycosides, aromatic cyanogenic glycosides, and others. This method and the chemical material findings may provide useful information for quality control when producing THSWD and related individual herbs, despite the ingredient complexity and specificity for TCM. The results can be used to develop a quality standard of the TCM compound THSWD and provide a theoretical basis for further study of THSWD metabolism *in vivo* to clarify the material basis and mechanism of its clinical application.

## Data Availability Statement

The raw data supporting the conclusions of this article will be made available by the authors, without undue reservation, to any qualified researcher.

## Ethics Statement

All experiments were subject to approval by the Committee on the Ethics of Animal Experiments of Anhui University of Chinese Medicine.

## Author Contributions

XD is responsible for designing the experiment private. LP is responsible for article writing. QB is responsible for the experimental operation. DP is responsible for data analysis.

## Funding

This research was supported by the National Natural Science Fund Regional Innovation and Development Joint Fund Project (No. U19A2009), Anhui University Collaborative Innovation Project (GXXT-2019-043), the Anhui Provincial College Natural Science Research Key Project (No. KJ2019A0466), Excellent and Top Talents Program in Colleges and Universities (No. gxyq2019034), Anhui Provincial Key Laboratory of Traditional Chinese Medicine Compounds (2019AKLCMF03), Open Project of National Key Clinical Specialty (Traditional Chinese Medicine Surgery)(2019zdzk04) and the Natural Science Research Project of Colleges and Universities in Anhui Province (2019fyyb038).

## Conflict of Interest

The authors declare that the research was conducted in the absence of any commercial or financial relationships that could be construed as a potential conflict of interest.
